# Experiences of using the i-PARIHS framework: a co-designed case study of four multi-site implementation projects

**DOI:** 10.1186/s12913-020-05354-8

**Published:** 2020-06-23

**Authors:** S. C. Hunter, B. Kim, A. Mudge, L. Hall, A. Young, P. McRae, A. L. Kitson

**Affiliations:** 1 College of Nursing and Health Sciences, Flinders University, Sturt Road, Bedford Park, Adelaide, SA 5042 Australia; 2 Caring Futures Institute, Flinders University, Adelaide, SA Australia; 3 Centre for Healthcare Organization and Implementation Research, U.S. Department of Veterans Affairs, Boston, USA; 4 Department of Psychiatry, Harvard Medical School, Boston, MA USA; 5 Internal Medicine Research Unit, Royal Brisbane and Women’s Hospital, Brisbane, QLD Australia; 6 School of Clinical Medicine, University of Queensland, Brisbane, QLD Australia; 7 School of Public Health and Social Work, Faculty of Health, Queensland University of Technology, Brisbane, QLD Australia; 8 Nutrition and Dietetics, Royal Brisbane and Women’s Hospital, QLD, Brisbane, Australia; 9 School of Exercise and Nutrition Sciences, Faculty of Health, Queensland University of Technology, Brisbane, QLD Australia

**Keywords:** I-PARIHS, Implementation science, Implementation frameworks, Co-design, Knowledge translation

## Abstract

**Background:**

The integrated-Promoting Action on Research Implementation in Health Services (i-PARIHS) framework is an implementation framework that has been developed and refined over the last 20 years. Its underlying philosophy is that implementing research into healthcare practice is complex, unpredictable and non-linear which therefore requires a flexible and responsive approach to implementation. Facilitation is recognized as the central ingredient of this approach, and i-PARIHS now provides a Facilitation Guide with associated tools. This multiple case study of four implementation projects explored how the i-PARIHS framework has been practically operationalized by diverse implementation project teams.

**Methods:**

A co-design approach was used to elicit the experiences of four implementation project teams who used the i-PARIHS framework to guide their implementation approach. We conducted the established co-design steps of (i) setting up for success, (ii) gathering the experience, and (iii) understanding the experience. In particular we explored teams’ approaches to setting up their projects; why and how they used the i-PARIHS framework and what they learnt from the experience.

**Results:**

We found both commonalities and differences in the use of i-PARIHS across the four implementation projects: (i) all the projects used the *Facilitation Checklist* that accompanies i-PARIHS as a starting point, (ii) the projects differed in how facilitation was carried out, (iii) existing tools were adapted for distinct phases: pre-implementation, during implementation, and post-implementation stages; and (iv) project-specific tools were often developed for monitoring implementation activities and fidelity.

**Conclusions:**

We have provided a detailed overview of how current users of i-PARIHS are operationalising the framework, which existing tools they are using or adapting to use, and where they have needed to develop new tools to best utilise the framework. Importantly, this study highlights the value of existing tools from the published i-PARIHS Facilitation Guide and provides a starting point to further refine and add to these tools within a future Mobilising Implementation of i-PARIHS (or “Mi-PARIHS”) suite of resources. Specifically, Mi-PARIHS might include more explicit guidance and/or tools for developing a structured implementation plan and monitoring fidelity to the implementation plan, including recording how strategies are tailored to an evolving context.

## Background

Successfully implementing new evidence into clinical practice can be very challenging. Strong evidence alone is not sufficient to change practice; therefore, the field of implementation science seeks to provide various approaches, frameworks and theories to inform systematic and successful implementation [1]. Specifically, these approaches assist in teasing out why particular evidence is successfully implemented in one setting and not another [2]. Without these frameworks guiding implementation efforts, it becomes difficult to understand what elements influenced implementation success or failure [2].

Various researchers from a multitude of backgrounds have developed different approaches to plan, guide and evaluate implementation efforts, with recent research suggesting more than 100 approaches being used in implementation research [3].

One such framework, the integrated-Promoting Action on Research Implementation in Health Services (i-PARIHS) is a conceptual framework that aims to represent the dynamic interplay of factors that influence successful implementation [4]. i-PARIHS argues that successful implementation results from the facilitation of an innovation with the intended recipients in their contextual setting; the proposition of i-PARIHS can be seen in Table 1 [4]. i-PARIHS represents an evolution of the PARIHS framework [5, 6], responding to several criticisms of that framework by providing clearer theoretical underpinnings as well as practical tools and case studies to help clinicians and researchers operationalise the framework [4, 7].

**Table 1:** Overview of i-PARIHS

i-PARIHS	
SI = Fac^n^ (*I* + *R* + *C*)	
SI = Successful implementation	
Fac^n^ = Facilitation	
*I* = Innovation	
*R* = Recipients (individual and collective)	
*C* = Context (inner and outer)	

The i-PARIHS framework specifies core constructs and sub-constructs which influence successful implementation, and is explicitly underpinned by relevant theories of innovation, behavioural and organisational change and improvement and focuses on facilitation as the active ingredient [4]. i-PARIHS also holds an underlying philosophy that implementing research into healthcare practice is complex, unpredictable and non-linear. Therefore, i-PARIHS was developed with complex multi-disciplinary team-based interventions in mind [5]. To support this, facilitation is positioned as the “core ingredient” in relation to the other constructs and is specified as both a specific role (“being” a facilitator) and a set of actions (“undertaking” facilitation).

In addition to this revised theory and clearly specified framework elements, the developers of i-PARIHS provide several tools to operationalize i-PARIHS in practice, outlined in their Facilitation Guide [7]. This includes a clear description of facilitator attributes, skills and roles, outlining the *Facilitator’s Journey*; a *Facilitation Checklist* to support structured assessment of the framework constructs;, and a *Facilitator’s Toolkit* to guide action.

Harvey and Kitson [4] outline how the *Facilitator’s Journey* develops, as facilitators begin as novices and work their way through to an experienced facilitator and finally an expert facilitator. Not only are there differences between novice, experienced and expert, but also there are differences between an internal and external facilitator [8]. For example, an individual can be an external novice facilitator, a stranger to the site in which the facilitation is occurring who will need to rapidly form relationships to tap into tacit knowledge about the local context and recipients. Alternatively, an existing staff member may take on the facilitator role in their own workplace, making them an internal novice facilitator, who may be familiar with the local context but may have to reframe their role from “doing” to “enabling”. The *Facilitation Checklist* provides users with various reflective questions to consider when working through and assessing the other i-PARIHS constructs (the innovation, recipients and context) [4] to inform tailoring of implementation strategies. The *Facilitator’s Toolkit* guides the facilitator through four action steps based in quality improvement and audit and feedback methods: clarify and engage, assess and measure, act and implement, and review and share.

Facilitation is what makes the i-PARIHS framework unique and it makes the framework flexible in its application, by encouraging iterative tailoring to a dynamic context. However, harnessing this flexibility requires an understanding of the core constructs in the i-PARIHS framework, as well as the complex potential roles and activities of a facilitator, which may be challenging for the novice facilitator to navigate [9]. Although the new resources included in the i-PARIHS Facilitation Guide offer assistance, their use in practice has not yet been described.

The purpose of the current study was to provide practical, real-world case studies on how implementation project teams have utilised and operationalised the i-PARIHS framework and its associated tools in their implementation efforts. By describing and comparing implementation projects undertaken in different contexts and by different groups, we aimed to illustrate how i-PARIHS tools can be applied in practice, and identify areas for further development to provide practical and accessible tools for clinical and academic implementation project teams. We were particularly interested in exploring how to balance the flexibility of the framework with the need to provide explicit direction or guidance for facilitators and researchers. Through exploring how the framework has been used, we aimed to generate guidance on how to operationalise the framework. Consistent operationalisation of the framework could also assist in evaluating the utility of the framework across different implementation efforts.

This exploratory study is the preliminary phase for a larger body of work that seeks to adapt the i-PARIHS framework into a suite of practical and pragmatic resources (called the Mi-PARIHS Project - **M**obilising **I**mplementation of **i-PARIHS**). Specifically, this study employed a multiple case study design that explored the following research question – how is the i-PARIHS framework operationalized by various implementation project teams?

## Methods

Using a co-design approach, the Mi-PARIHS team (SCH, ALK) collaborated with four implementation project teams (lead by BK, AM, PM, LH, AY) to understand their views and experiences of how they used i-PARIHS in their implementation projects. Co-design is a methodology that focuses on bringing together end users’ experience in a collaborative process [10]. Authentic collaboration involves engaging these users in all phases of the research process [11]. Therefore, this study was a collaboration between the Mi-PARIHS team and implementation project teams who had used the framework in their work and who had agreed to share their experience and tools to inform ongoing development and refinement of i-PARIHS resources. The four implementation project teams included researchers, clinicians and facilitators; had all used i-PARIHS (amongst others) as a framework for implementation; and had adapted resources and tools to operationalise the framework.

The included implementation project teams were selected as a convenience sample reflecting a range and breadth of research teams known to ALK who were using i-PARIHS and who agreed to be part of the case study. These teams were selected to meet the following criteria:

- Use of i-PARIHS as an implementation framework
- Geographical spread
- Complex interventions involving multidisciplinary teams
- Diversity in project size and clinical specialism

No results from the studies are shared in this paper, as the focus is not on the outcomes of their implementation, but rather, the experience of using the i-PARIHS framework as their implementation framework. In order to collect information around their use of i-PARIHS, we utilised the experience-based co-design toolkit [10]. This toolkit outlines 5 steps for co-design - 1) set up for success, 2) gather the experience, 3) understand the experience, 4) improve the experience and 5) monitor and maintain the experience. In the current study, we used the first three steps.

Step one, *set up for success,* involves understanding what will contribute to success and then planning and investing effort into this. For this study, we spent considerable time on identifying the common motivations for this project and how we collectively agreed upon a co-design approach to value each member’s theoretical and/or experiential expertise with the framework, as opposed to a researcher/participant relationship. Two initial in-person planning meetings were held between the Mi-PARIHS team and the implementation project teams (one in Australia and one in the USA). Subsequent team meetings were held between all partners via Zoom software [12] to collectively establish the data collection and analytic approach.

Step two, *gathering experience*, involves getting a sense of what the current experience is. This study used informal interviews and user stories to gain insight on each implementation project team’s experiences of using i-PARIHS. Each team wrote their approach to using the framework into a structured case summary template and had one-on-one meetings with author SCH to discuss in more detail, via Zoom software. Collectively, representatives from each of the four implementation projects included in our case study systematically worked through this information, with SCH and ALK leading the consolidation and interpretation and other team members suggesting changes and improvements via further video conference meetings and email correspondence. The resulting refined information was then mapped onto the framework and checked for relevance and sense-making with the entire authorship team.

Step three, *understanding the experience*, involves taking the knowledge to stimulate further discussion and dialogue. Therefore, SCH and ALK emailed the final case summaries back to the implementation project teams and as a group the entire authorship team developed cross-case analyses and overall conclusions and implications via regular email correspondence and refining drafts and where necessary, phone conversations and video conference meetings. Through this process we were able to identify which dimensions of the framework are consistently used and how, compared with what is optionally used, and what may be missing from the framework.

## Results

Up to two members each from four implementation project were included in this case study. These implementation projects include three from Australia and one from the United States of America. These projects include:

- BHIP: **B**ehavioural **H**ealth **I**nterdisciplinary **P**rogram [13]
- CHERISH: **C**ollaboration for **H**ospitalised **E**lders **R**educing the **I**mpact of **S**tays in **H**ospital [14].
- The SIMPLE Approach: A **S**ystematised, **I**nterdisciplinary **M**alnutrition **P**athway for imp**L**ementation and **E**valuation in hospitals [15].
- REACH: The **R**esearching **E**ffective **A**pproaches to **C**leaning **H**ospitals study [16]

The following case summaries explore how each implementation project utilised and operationalised the i-PARIHS framework. The four selected implementation projects represent the fields of mental/behavioural health, public health, multidisciplinary acute care and nutrition care, and include a quality improvement project (SIMPLE), a combined quality improvement project and randomised implementation trial (BHIP), and randomised controlled trials (CHERISH and REACH). They also varied in the setting with one outpatient mental health care project (BHIP) and three acute care projects (CHERISH, SIMPLE and REACH). Table 2 provides a high-level overview of each implementation project in order to provide background information, Table 3 provides project specific detail, and Table 4 provides detail on the tools adapted or developed for each project. These three tables provide the information necessary to understand the case summaries and analysis, and further details regarding each project are available through their respective project-specific publications (BHIP – [13, 17]; CHERISH – [14, 18]; SIMPLE – [15, 19]; REACH – [16, 20, 21]).

**Table 2:** Summary of Each Implementation Project

Project	Problem	Innovation	Facilitation	Recipients	Context
**1 BHIP**	Limited delivery of anticipatory, patient-centred, and coordinated outpatient mental health care.	Collaborative Chronic Care Model (CCM) for interdisciplinary team-based care in general mental health clinic settings.	Internal-external model of facilitation adapted from Kirchner et al. (2014) and based on i-PARIHS	Interdisciplinary general mental health care team members (e.g. clerk, nurse, psychiatrist, psychologist, social worker) at nine U.S. Department of Veterans Affairs (VA) medical centres.	**Outpatient mental health care setting**
					*Outer context*
					In 2013, VA leadership launched a nationwide initiative to establish interdisciplinary teams in each VA medical centre throughout the United States. In 2015, the VA adopted the CCM and partnered with the study team to develop CCM implementation support.
					*Inner context*
					The inner context varied by medical centre.
**2 CHERISH**	High prevalence of geriatric complications (e.g. delirium, functional decline) in older inpatients	A multicomponent intervention to reduce complications and improve outcomes	Internal-external model of facilitation based on i-PARIHS	Interdisciplinary acute care team members (nurses, allied health professionals, medical staff)	**Acute care setting**
					*Outer context*
					New national standard on comprehensive care and delirium clinical care standard created an impetus for improving care of older patients.
					*Inner context*
					The inner context varied by hospital and ward.
**3 SIMPLE**	Suboptimal and inefficient management of malnutrition in hospitals	Systematised Interdisciplinary Malnutrition Program Implementation and Evaluation – for enabling a system and team approach to better management of malnutrition	Internal-external model of facilitation based on i-PARIHS	Multidisciplinary teams and dietetics departments in six publicly funded hospitals in Australia	**Acute care setting**
					*Outer context*
					State-wide roll-out of electronic medical
					records exposed gaps in systems of malnutrition care and unsustainable demand on dietetics services.
					*Inner context*
					The inner context varied by hospital, ward and dietetics department.
**4 REACH**	Inconsistent cleaning practices in hospitals despite detailed cleaning guidelines	An environmental cleaning bundle – a bundle of evidence-based practices to improve cleaning performance and reduce infections	External facilitation with local champions	Environmental services staff members employed in a role that included ward cleaning across 11 hospitals (public and private) in Australia.	**Acute care setting**
					*Outer context*
					National accreditation requirements and infection control guidelines required hospitals to have a comprehensive cleaning program.
					*Inner context*
					The inner context varied by ward.

**Table 3:** Project Specific Detail

	1 BHIP	2 CHERISH	3 SIMPLE	4 REACH
**Research Team**	Mental health services researchers and implementation scientists from the VA Behavioural Health Quality Enhancement Research Initiative (QUERI) Program.	A collaborative national team of clinical and academic researchers with an international advisor. This team included geriatric content experts, effectiveness evaluation experts, and implementation experts.	A collaborative state-wide team of clinicians and clinical and academic researchers with an international advisor.	A collaborative national team of researchers. This team included implementation science experts, infection control nursing experts, epidemiology experts, psychology experts, medical microbiology experts, and economics and statistics experts.
**Funding**	This project was funded as a part of the VA Behavioural Health QUERI Program (Grant # QUE 15–289), which was competitively funded from 2015 to 2020.	This project was funded by a Queensland Accelerate Partnership Grant (co-funded by Queensland Government, Queensland University of Technology and participating health services) from 2015 to 2017.	This project was funded for implementation by the Allied Health Professions Office of Queensland; evaluation was funded through an Australian Centre for Health Services Innovation (AusHSI) Implementation Grant.	This project was funded by a National Health and Medical Research Council (NHMRC) Partnership Project.
**Study aims**	(i) To assess whether the evidence-based CCMs can be successfully implemented using existing staff in general mental health clinics supported by internal and external implementation facilitation (ii) To evaluate the impact of CCM implementation efforts on patient health status and perceptions of care	(i) To evaluate the effectiveness and cost-effectiveness of the Eat Walk Engage program for inpatients aged 65 years and older. (ii) A process evaluation to understand how and where the program worked.	(i) To implement SIMPLE in six pilot hospitals across Queensland, purposively sampled to ensure diverse service models and case mix. (ii) To evaluate whether SIMPLE delivered more appropriate nutrition care to more patients at a lower cost per patient.	(i) Evaluate the effectiveness of an environmental cleaning bundle to reduce hospital acquired infections in Australian hospitals (ii) Estimate the cost-effectiveness of a decision to adopt the environmental cleaning bundle for Australian hospitals.
**Method**	Combined research and quality improvement project. This project utilised a randomised stepped-wedge implementation trial – Hybrid II design: Concurrent measurement of intervention effectiveness and implementation effectiveness.	Cluster randomised controlled trial - Hybrid I design: Primary measurement of intervention effectiveness, cost-effectiveness with pre-planned measurement of implementation effectiveness.	Pre-post audits of nutrition care practices and dietetics occasions of service was used to evaluate the implementation of SIMPLE.	Randomised control trial using a cross-sectional stepped-wedge randomised allocation.
**Implementation approach**	*What did they do*: Internal-external model of facilitation. *Who did it*: A study-funded external facilitator with a site-funded local internal facilitator.	*What did they do*: Internal-external model of facilitation. *Who did it*: Study-funded external facilitators and locally recruited clinical internal facilitators at each site. Partnership funding meant that the health service indirectly funded their novice facilitators	*What did they do*: Internal-external model of facilitation, with Consolidated Framework for Implementation Research (Damschroder et al., 2009) used to describe the baseline context at each site (using interviews with key informants) *Who did it*: Study-funded external/experienced facilitators across the sites and study-funded locally recruited clinical internal/novice facilitators at each site.	*What did they do*: External model of facilitation with local champions. *Who did it*: The research team assessed local context and provided support across the sites with local change champions in each site.
**Evaluation Measures**	Team functioning, team processes, provider interviews for care experiences reflecting CCM [implementation outcomes]; patient surveys for health status and perceptions of care (at three time points), mental health hospitalization rate [intervention outcomes].	Ward process measures, patient interviews, evaluation of context, recipients, facilitation process and multi-disciplinary team engagement.	Nutrition care practices (documented and patient-reported), dietetic and allied health assistant occasions of service, evaluation of context, and facilitation process.	Pre and post questionnaire to measure knowledge and attitudes in staff, changes in practice (pre-post bundle alignment) to assess intervention fidelity, improvements in cleaning performance as assessed through routine collection of gel dot audits.
**Scale**	9 sites.	8 wards (4 control and 4 intervention wards) across 4 sites.	6 sites.	11 acute public and private Australian hospitals.
**Implementation Duration**	12 months per site.	18 months.	6 months.	4–12 months.

**Table 4:** Tools Adapted or Developed for Each Implementation Project

	1 BHIP	2 CHERISH	3 SIMPLE	4 REACH
**Pre-implementation**	External facilitators (part of implementation project team) adapted the *Facilitation Checklist* to a pre-implementation tool to assess the baseline inner context, recipients, and available resources to support facilitation using framework constructs.	External (expert) facilitators (part of the implementation project team) used the *Facilitator’s Journey* for recruitment and training of internal novice facilitators External facilitators adapted the *Facilitation Checklist* and guided the internal (novice) facilitators to assess inner context and recipients using framework constructs	External (expert) facilitators (part of the implementation project team) used the *Facilitator’s Journey* used for recruitment and training of internal novice facilitators External facilitators adapted the *Facilitation Checklist* which was used by some internal (novice) facilitators to assess inner context and recipients using framework constructs	The implementation project team (health service researchers and evaluators) adapted the *Facilitation Checklist* to assess context and recipients using framework constructs. The study team also developed a quantitative tool to rate baseline alignment against bundle (intervention characteristics), individual (intervention recipients) and site readiness (context).
**Implementation**	External facilitators used improvement progress log for formative evaluation, which was informed by the *Facilitation Checklist* to document and dynamically shape facilitation steps, complementing separate tools used to track (i) progress on CCM-concordant care process development/redesign and (ii) implementation site-facing external facilitation activities	External facilitators used the *Facilitator’s Toolkit* to design materials for facilitator training and mentoring throughout project Adapted *Facilitation Checklist* used at baseline was repeated by the internal facilitators to monitor progress and changes in recipients and inner context to iteratively adapt strategies *Facilitator’s Journey* informed external-internal facilitation model	External facilitators used the *Facilitator’s Toolkit* for facilitator training and mentoring at project commencement (adapted from those used in CHERISH) Adapted *Facilitation Checklist* used at baseline was repeated by some internal facilitators to monitor progress and changes in recipients and inner context *Facilitator’s Journey* informed external-internal facilitation model	The implementation project team developed an implementation plan template based on framework constructs. The research team worked with local staff to populate this at each site to create a tailored plan to address gaps identified during pre-implementation (i.e. low scores). The implementation project team used a monitoring tool to record progress against the plan and support implementation and local facilitation.
**Evaluation**	Evaluation codebook that reflects elements of the *Facilitation Checklist* and the *Facilitator’s Toolkit*, for use by the research team for analysing qualitative interviews focussed on interdisciplinary general mental health care team members’ experiences of implementing the CCM per its six core elements	*Facilitator’s Toolkit* informed process evaluation in collaboration between external facilitators and research team *Facilitator’s Journey* assessed through qualitative interviews by research team focussed on progression from novice to experienced facilitator	*Facilitator’s Toolkit* provided coding framework for qualitative interviews focussed on facilitation activities	Quantitative tool based on i-PARIHS constructs used by study team to re-assess changes in bundle alignment and success of implementation. Qualitative summary of barriers and enablers identified during monitoring, grouped according to overarching i-PARIHS constructs

### Case 1 – (BHIP)

This project, the **B**ehavioural **H**ealth **I**nterdisciplinary **P**rogram (BHIP) project, implemented the evidence-based Collaborative Chronic Care Model (CCM) [22–28] into care that is delivered by interdisciplinary teams in general mental health clinics. This model focuses on improving mental health care by enhancing the implementation of the CCM’s six core elements – work role redesign, self-management support, provider decision support, clinical information systems, community resources and organizational/leadership support [17]. This model requires flexible implementation and tailoring to local context, therefore is a complex intervention to implement. This project implemented the model in mental health clinics situated within nine Department of Veteran Affairs (VA) medical centres across the United States. Table 3 provides an overview of the project and further details can be obtained in the project specific publications [13, 17].

The project used Kirchner et al.’s [8] internal-external facilitation model to implement CCM, and i-PARIHS was chosen because it is a framework that explicitly identifies facilitation as the construct that activates the other constructs toward successful implementation. In addition, the project used the four stages (pre-conditions, pre-implementation, implementation and maintenance and evolution) of the Replicating Effective Programs framework to organise their facilitation into different stages [29].

#### Implementation approach

This project used i-PARIHS to guide their internal-external facilitation approach. This approach involved each site having a study-funded experienced/expert external facilitator (bringing intervention/implementation content and process redesign expertise) working with a site-funded novice/experienced internal facilitator (bringing knowledge and experience of local culture, structures, and policies) for 1 year. The external facilitators had knowledge of the i-PARIHS framework and the specific constructs that play a part in implementation. The external facilitators utilised this knowledge to plan and conduct their interactions with each internal facilitator and site.

The goal for the one-year implementation period per site was for the external facilitator to train and support the internal facilitator around CCM content and implementation, with the plan for the internal facilitator to develop the appropriate skills to serve as the local expert on the intervention beyond the one-year implementation period. Specifically, the year started with the external and internal facilitator having weekly phone calls for real-time coaching and problem-solving, which tapered as the year progressed (and as the internal facilitator developed facilitation expertise) to be held on an as-needed basis. The internal facilitators also participated in a monthly learning community call to share their facilitation experiences and seek input from fellow internal facilitators on how best to respond to various implementation-related situations that they face. The implementation project team coordinating these external-internal facilitator and monthly group calls were guided by i-PARIHS, but no formal curricular materials based solely on i-PARIHS were used to steer these calls.

In addition to the study-funded external facilitator and the site-funded internal facilitator, the implementation at each site involved interdisciplinary general mental health care team members (e.g., clerk, nurse, psychiatrist, psychologist, social worker) and their clinic leadership as champions when possible.

The facilitation approach aligned closely with the i-PARIHS framework and the *Facilitation Checklist* and the *Facilitator’s Toolkit* were closely drawn on in developing the facilitation approach.

Table 4 outlines the three main i-PARIHS based tools that this project developed. The pre-implementation assessment tool utilised the *Facilitation Checklist* [7] and adapted the questions relating to inner context and recipients, as the purpose of this phase was to obtain contextual information from site-based stakeholders. The pre-implementation assessment tool was utilised by the external facilitators as a conversation guide to obtain contextual information from site-based stakeholders (e.g., leadership, frontline providers). This tool focused mostly on assessing the baseline facilitation, recipient and context constructs as they pertained to each site. Given the information came from the site-based stakeholders, this tool did not focus on details regarding the innovation (i.e., CCM for interdisciplinary team-based care in general mental health clinic settings).

For the monitoring phase, an improvement progress log for formative evaluation was developed based on the *Facilitation Checklist* [7]. This improvement progress log was utilised by the external facilitators to document and dynamically inform facilitation steps through the year-long active implementation period. This complemented two non-i-PARIHS-specific monitoring tools: (i) a CCM process summary that documented specific CCM-concordant care processes that were designed/redesigned throughout implementation and (ii) a time-motion tracker [30] that logged how external facilitators spent their time on facilitation activities.

For the evaluation phase, an evaluation codebook is currently being finalised which focuses on all of the i-PARIHS constructs and sub-constructs and closely reflects the elements of the *Facilitation Checklist* and *Facilitator’s Toolkit* [7]. The codebook is to be used by the implementation project team to analyse qualitative interviews conducted with the interdisciplinary general mental health care team members, which focused on the members’ experiences of implementing the CCM per its six core elements of work role redesign, self-management support, provider decision support, clinical information systems, community resources and organizational/leadership support.

#### Scalability and sustainability

Using the i-PARIHS framework directly contributed to the project’s ability to scale up and sustain the intervention. The detailed documentation of facilitation activities from the study, particularly in consideration of the i-PARIHS contextual constructs (e.g., planned regular communication with leadership and other stakeholders at implementation sites), was used as an implementation roadmap that helped train and guide additional external facilitators who were taking part in scaling up and spreading CCM-based care to additional VA medical centres beyond the nine-site trial reported on in this paper [31]. In terms of sustainability, the goal was to have the external-internal facilitation model help ensure that content/redesign skills were transferred to and sustained at the implementation sites.

### Case 2 – (CHERISH)

The **C**ollaboration for **H**ospitalised **E**lders **R**educing the **I**mpact of **S**tays in **H**ospital (CHERISH) project implemented “Eat Walk Engage”. This ward-based program engages interdisciplinary teams in acute care wards to support adequate nutrition and hydration, early and graded mobilisation and meaningful cognitive engagement. These are evidence-based non-pharmacological delirium prevention strategies, and pilot data showed improved care processes and reduced length of stay [32]. This multidisciplinary program requires flexible implementation and tailoring to local context and recipients, therefore is a complex intervention to implement. CHERISH evaluated implementing this program into four hospitals across Queensland, Australia. Table 3 provides an overview of the project and further details can be obtained in the project specific publications [14, 18].

The original PARIHS framework had been applied retrospectively to describe development of the pilot program Eat Walk Engage [32]. In the CHERISH study, i-PARIHS was used prospectively. Facilitation was recognised as the central component of the program, and the CHERISH study adopted a facilitation model of expert external and novice internal facilitators for expanding the successful pilot to other hospitals.

#### Implementation approach

This project used i-PARIHS to inform an internal-external facilitation approach. Implementation project team members AM and PM worked as external/expert facilitators with intervention content and implementation expertise. The internal/novice facilitators were selected through a recruitment process informed by the *Facilitator’s Journey* and provided with explicit training on intervention content as well as implementation training using i-PARIHS constructs and the *Facilitator’s Toolkit* [7]. The external facilitators were supported by an implementation steering group which included implementation academics, clinical leaders, a consumer and the internal facilitators. The internal facilitators (one nurse, two dietitians and one occupational therapist) were supported by the external facilitators to undertake an initial context and recipient mapping, build a team/workgroup, network with stakeholders, identify improvement goals and use Plan, Do, Study Act (PDSA) small cycles [5] to achieve improvements. The internal facilitators were supported through monthly 2-h group face-to-face meetings with the external facilitators, which included didactic content about the program aims, implementation science and quality improvement methods as well as guided reflection about their local context and progress. External facilitator PM was available for ad hoc discussions and attended some team and one-on-one meetings with the internal facilitator at their site. Support gradually moved from the external facilitators to more peer mentoring as they developed their skills and confidence.

Table 4 outlines the three main i-PARIHS based tools that this project developed. In the pre-implementation phase, this project utilised the framework to develop phased, project-specific training resources to support and train the internal facilitators. Recipient and context assessments based on the *Facilitation Checklist* [7] were utilised in the pre-implementation phase to help shape the planning of facilitation and provide baseline data. The external facilitation team developed a custom-designed reporting tool which allowed facilitators to rate features of the recipients and context on a − 2 (strong barrier) to + 2 (strong enabler) scale in addition to providing qualitative comments. This graded tool supported reflection with the external facilitators and peer internal facilitators, assisting to visually identify key barriers and enablers and tailor implementation to each ward. For the monitoring phase, the recipient and context assessments were repeated twice in order for facilitators to reflect on progress, identify any changes, learn from successes and continue to actively tailor their strategies. The implementation project team also measured process indicators of ward-based care related to the primary program goals (nutrition care, mobility and meaningful engagement). The external facilitators manually recorded strategies used by the internal facilitators at each site using meeting minutes and field notes, as a measure of intervention “dose” to contribute to evaluation of this multi-site study. This implementation project team also engaged one of the PARIHS/i-PARIHS developers (Gill Harvey) to conduct qualitative interviews which were focused on describing and understanding the novice facilitators’ journey.

#### Scalability and sustainability

This project explicitly tested scaling and spread of a successful pilot model, clearly specifying the additional resources and expertise required to implement the Eat Walk Engage program in new sites. The use of the i-PARIHS framework assisted in training and mentoring internal/novice facilitators to become experienced facilitators, aiming to support local sustainability and scale up beyond the implementation period. It also helped to understand variable success between sites and identify recipient and contextual features that might help or hinder implementation success in future sites. By demonstrating the feasibility of scale and spread to new sites, documenting realistic implementation resource and clinical resource requirements, and demonstrating successful outcomes, the program has been able to secure recurrent funding from the Queensland Health Department to expand Eat Walk Engage to additional hospitals. In terms of spread and sustainability, the model continues as an external-internal facilitation model to help ensure that intervention content and implementation skills are developed and sustained at sites.

### Case 3 – SIMPLE

This project, a **S**ystematised, **I**nterdisciplinary **M**alnutrition **P**athway for imp**L**ementation and **E**valuation in hospitals (SIMPLE) implemented an interdisciplinary and systematic approach to malnutrition management for hospital inpatients (the SIMPLE approach). The SIMPLE approach involves supportive nutrition intervention being provided from the time of risk identification, with dietetic review for those people with complex nutrition needs or where needs are not met by the supportive interventions. This is different to standard practice in Australia, where those people identified at risk of malnutrition are provided with nutrition assessment and individualised care planning by the dietitian which then prompts nutrition intervention. Malnutrition is a “wicked” problem that requires multi-dimensional and flexibly delivered intervention strategies, therefore making this project a complex intervention to implement [25]. This project implemented the SIMPLE approach into six hospitals across Queensland. Table 3 provides an overview of the project and further details can be obtained in the project specific publications [15, 33].

This project utilised the i-PARIHS facilitation model, as the implementation project team considered the facilitation component of i-PARIHS to be key to enabling change in local teams required to successfully implement SIMPLE. Additionally, AY was involved in the CHERISH implementation project team and could see that the facilitation approach used in this project could be applied to SIMPLE.

#### Implementation approach

This project used i-PARIHS to inform an internal-external facilitation approach. A facilitative approach was taken in order to enable local teams to develop and implement strategies that aligned with the SIMPLE approach, which was anticipated to require complex systems, process and/or workforce redesign across disciplines and hospital departments. Each intervention site (6 hospitals across Queensland, diverse case-mix and locations) had a local clinician acting as an internal/novice facilitator with 0.2 FTE funded time for 9 months (6 months implementation, and additional 3 months for training and evaluation). The internal facilitators were selected through a recruitment process informed by the *Facilitator’s Journey* [7], and were provided with training on intervention content as well as in stakeholder engagement, context assessment and implementation (2 × 1-h sessions based on the i-PARIHS *Facilitator’s Toolkit*). Three of the six internal facilitators attended a Knowledge Translation three-day course which had a significant component on facilitation and i-PARIHS.

The internal facilitators were supported via tele-monitoring with peers and external/experienced facilitators in monthly meetings plus phone and email support. These sessions were used to reflect on progress and provide advice and suggestions (usually around context, either helping them articulate what was happening in the context and how that was influencing progress or prompting them to gain a better understanding of the context).

The Consolidated Framework for Implementation Research (CFIR [34],) was used to conduct the pre-implementation context assessments. However, some of the internal facilitators used the i-PARIHS *Facilitation Checklist* questions to assist with their local ward context assessments, although this was not mandatory.

Table 4 outlines the four main i-PARIHS based tools that this project utilised. Unlike the other projects that utilised tools to plan, monitor and evaluate overall implementation, this project developed tools mostly to support and guide the facilitators (this was due to the use of i-PARIHS for facilitation, and the CFIR framework for pre-implementation context assessment), as well as to evaluate the facilitator role. In the pre-implementation phase, this project utilised the *Facilitator’s Journey* to inform and develop “Expressions of Interests” to recruit facilitators [7]. i-PARIHS also informed initial training for the facilitators using the *Facilitator’s Toolkit* [7]. For the pre-implementation phase, recipient and context assessments were utilised based on the *Facilitation Checklist* [7] to help shape the planning of facilitation. For the monitoring phase, the recipient and context assessments were repeated by some facilitators to reflect on progress, any changes, learn from successes and continue to actively tailor their strategies. Finally, for the evaluation phase, the *Facilitator’s Toolkit* [7] was utilised to analyse qualitative interviews with facilitators to gain an understanding of what they did and the challenges they faced. With the exception of this qualitative coding framework, all tools were adapted from those used in (CHERISH). The implementation project team member AY reflected that having pre-existing tools available as templates helped to see how the framework could be operationalized for SIMPLE.

Unlike some of the other projects, this project used i-PARIHS in addition to the use of a primary framework (CFIR). The implementation project team member AY identified that it would have been useful to have the facilitators use i-PARIHS in a more structured way to guide implementation throughout the project and repeated on conclusion to evaluate overall success, however this was not done due to the initial use of CFIR for context assessments and time constraints on the facilitators.

#### Scalability and sustainability

Whilst evaluation of the facilitator experience identified the benefits of using the i-PARIHS facilitation model and framework [19], ongoing roll-out of the SIMPLE approach across Queensland hospitals is using an unfunded local champion model supported by a centralised facilitation team. Lack of ongoing external funding for the facilitator role in addition to the beliefs of decision makers that local health services should redirect or invest internal resources to implement changes to care delivery has meant that the dedicated facilitator role has not continued. Evaluation of these two different facilitation models (i.e. unfunded champions vs funded facilitators) to implement the same intervention could generate further insight into the facilitation process.

### Case 4 – REACH

This project, the **R**esearching **E**ffective **A**pproaches to **C**leaning **H**ospitals (REACH) project, implemented an evidence-based environmental cleaning bundle. Despite detailed cleaning guidelines, implementing and sustaining effective cleaning programmes in hospitals is challenging. Therefore, this project focused on a bundle which is a set of evidence-based practices that when performed collectively and reliably have proven ability to improve patient outcomes. Given this intervention requires a bundle of evidence-based practices, it is a complex intervention to implement. This project implemented this intervention within 11 hospitals across Australia. Table 3 provides an overview of the project and further details can be obtained in the project specific publications [16, 20, 21].

Within this project, i-PARIHS was used due to the chief investigator LF having familiarity with the framework. In an initial pilot study [35] the team developed the cleaning bundle and tested the implementation and tools for the intervention in one hospital. In this pilot, PARIHS (the original iteration of i-PARIHS, 5) was used prospectively as the implementation framework. Based on this successful experience, the REACH trial then used the newly developed i-PARIHS framework to guide implementation. Further, i-PARIHS was selected as a suitable implementation framework due to the intervention needing to be tailored to each site. The intervention had core components that were non-negotiable for effectiveness in addition to adaptable components. The implementation project team selected i-PARIHS as a way of supporting sites in identifying the extent of practice change required to meet the core requirements and in tailoring these adaptable components to the local context.

#### Implementation approach

This project did not formally identify facilitator roles but rather informally drew on the process of facilitation to identify local staff (not paid to take on this role) who could be used as local change champions and undertake the site-specific responsibilities of the study co-ordination. These champions were identified within each site and these champions were the point of contact to ensure the intervention was continuing successfully. Initially, these individuals were utilised to ensure data quality for the research, but they ended up working informally in a facilitative capacity. Having these site teams allowed the researchers to maintain regular communication with each site and provide support, and also allowed for the opportunity to work through any issues as they arose.

In addition, this implementation project team used i-PARIHS to map the hospital characteristics and context, current infection prevention policies and practices and conduct surveys. This information was used to develop a tailored implementation strategy for each site, informed by behavioural change and adult learning theories. Throughout the trial, these aspects were regularly monitored, with communication between the central study team and local site team to address emerging issues and provide relevant support to ensure success.

Table 4 outlines the main i-PARIHS based tools that this project utilised. This project took a systematic approach to utilising the framework; they systematically worked through the constructs of the framework and adapted them to how they specifically related to their intervention, resulting in an “i-PARIHS to REACH” tool. This tool works through the i-PARIHS *Facilitation Checklist* [7] and adapts the questions to specific REACH questions (Table 5). This project then used this to develop an implementation toolkit to assist in tailoring the intervention to each site which included a structured pre-implementation assessment tool, a monitoring tool and an evaluation tool. The pre-implementation assessment tool assessed bundle compliance (intervention characteristics), context (inner and outer) and recipients (based on the *Facilitation Checklist*, 7) and rated these in relation to the degree of alignment (reality vs optimal) out of 1 (low) to 5 (high). Issues that would impact readiness to implement the intervention were noted qualitatively. This assessment tool (through the grading system) allowed the implementation project team to produce visual representations so they could easily identify where each site was in relation to the constructs.

**Table 5:** Example of REACH Adaptation of i-PARIHS *Facilitation Checklist*

Elements & key questions – i-PARIHS	REACH questions	Prompts
**1. Characteristics of the cleaning bundle intervention**		
**Who it affects?** *Who is likely to be affected by the proposed innovation?*	Who is directly impacted?	Composition, roles and responsibilities of site team Environmental services workforce
**Underlying knowledge sources** *Is the evidence viewed as rigorous and robust? Is there a shared view about the evidence? What other evidence is important at this site?*	How is the bundle perceived?	Is there a shared view about the evidence? What other evidence is important at this site?
**Clarity** *Is the evidence packaged in an accessible and usable form? Will people be able to see easily and clearly what is proposed in terms of practice?*		
**Degree of fit (compatibility or contestability)** *How well does it ‘fit’ the local setting? Is it likely to be accepted or contested by those people who have to implement it?*	How does the bundle align with current practice at the site?	What is the extent of change required to implement? *Refer to completed intervention gap analysis*

The monitoring tool was a questionnaire and related specifically to monitoring the uptake of the intervention components and progress against the agreed implementation strategy. Each site regularly (approximately every 2 months) completed this questionnaire. The monitoring tool was not used for process evaluation per se, rather to examine the implementation process and any emerging contextual barriers, to identify any issues that needed to be addressed proactively.

Finally, the evaluation tool was an adapted version of the pre-assessment template focussing on scoring bundle alignment (intervention characteristics) in order to provide a final snapshot of the sites’ performance. In addition to this qualitative information relating to barriers and enablers (context and recipients) identified during monitoring were also summarised for inclusion in handover reports.

#### Scalability and sustainability

The systematic and structured approach to implementation taken within this project was identified as useful for scalability. The local engagement and local ownership of the intervention also resulted in more sustainable outcomes, including improvements in staff knowledge and attitudes, improvements in cleaning performance and reductions in infection rates. Further, at the time of project wrap up, the implementation project team had a final meeting with each site to discuss their plan for moving forward. Sites were provided with a detailed report on what they had accomplished, and what gaps remained. This formal handover provided them with complete ownership of the intervention moving forward.

## Discussion

The purpose of this paper was to elicit the real-world experiences of four implementation project teams that had used i-PARIHS as their implementation framework to inform the future development and refinement of a range of validated resources and tools that could be used in implementation projects. Our goal within this paper was to provide practical, real-world case studies on how implementation project teams have utilised and operationalised the framework in their implementation efforts. We examined how current users of i-PARIHS are operationalising the framework, which existing tools they are using, and where they have needed to develop new tools.

We found both commonalities and differences in the use of i-PARIHS across the four implementation projects, making clear that i-PARIHS (like many implementation frameworks) is a guide and not a recipe. All implementation project teams had chosen i-PARIHS because of its suitability for implementing complex healthcare interventions in different sites, where there is a predictable need to adapt or tailor strategies to diverse contexts. Existing i-PARIHS resources, the *Facilitator’s Toolkit* and the *Facilitation Checklist*, published by Harvey and Kitson [7], were relied upon by all four implementation project teams. The *Facilitator’s Toolkit* provides a model for facilitation and the *Facilitation Checklist* provides prompting questions and things to consider for each of the i-PARIHS constructs. The consistent use of these resources across the implementation project teams demonstrates the central place of facilitation and the value of providing structured support. However, each implementation project modified and adapted these resources to their unique project needs, sometimes aligned with other conceptual frameworks, highlighting the need for local tailoring. For example, most implementation project teams modified the *Facilitation Checklist* by selecting specific sub-elements relevant to their project, and modifying wording (as seen in the “i-PARIHS to REACH” tool, Table 5). The CHERISH and REACH projects adapted the *Facilitation Checklist* by providing a simple ordinal scoring system to indicate which sub-elements were considered stronger barriers and enablers, allowing simpler visualisation of complex information. The *Facilitation Checklist* also contributed to project evaluation by informing coding of qualitative interviews in BHIP and SIMPLE, and by informing an ordinal scoring system to indicate responses to implementation in REACH. One project (SIMPLE) learned from another (CHERISH) and was able to adapt tools to suit their needs.

Information from using the *Facilitation Checklist* helped to guide implementation of each project, allowing adaptation or tailoring of planned strategies. How this actually occurred was less explicit. The REACH project developed an implementation plan template which assisted in tailoring the intervention to each site. However, the other three projects utilised a more iterative approach to implementation, supporting their internal/novice facilitators to adapt their strategies to an evolving context, under guidance of external/expert facilitator.

The range of ways in which facilitation was operationalised, including the length of time allocated to the implementation itself, also varied across the projects. The *Facilitator’s Toolkit* was used to train and mentor novice facilitators in the CHERISH and SIMPLE projects using formal curriculum sessions, and both projects provided regular opportunities to share progress in their facilitation activities with their peers under the guidance of an expert facilitator, emphasising the importance of experiential learning. Two projects acknowledged the *Facilitator’s Journey*, and specifically recruited internal novice facilitators, supported by expert facilitators who provided regular mentoring and opportunities for reflection. CHERISH and SIMPLE used the role descriptions from the Facilitation Guide [5] to recruit and train facilitators. Variability was seen in how the facilitation was funded/resourced, and also in how each implementation project team considered sustainability, scalability, and capacity building beyond the period of active implementation.

In addition to the facilitator role, there was diversity in implementation project team composition and roles in these projects. Some of the projects included researcher/facilitators, others were researcher/evaluators and others were researcher/facilitator/clinical experts. These different users are likely to have different support requirements. Those enacting the facilitation role are likely to value practical tools to plan, guide and tailor their implementation strategies, while those in an evaluation role may be more interested in reliable tools to record the context, implementation and impact measures, and the implementation experience.

In addition to the variations identified by this study in how i-PARIHS has been operationalised, common elements were also identified. All of the projects adapted the existing tools to support three distinct stages of implementation: 1) pre-implementation (diagnostics and planning) 2) during implementation (guiding and monitoring) and 3) post-implementation (evaluating). The *Facilitation Checklist* was adapted in all projects to provide a baseline assessment, and was also used in monitoring and/or evaluation. The *Facilitator’s Toolkit* was explicitly used for facilitator training in two projects, and guided part of the evaluation in three projects. The *Facilitator’s Journey* was clearly integrated into two projects which used an expert external and novice internal facilitator. However, the link between planning and doing was not explicitly supported by existing tools, nor was monitoring implementation fidelity, so that each team had to develop their own approach. Tools that could guide development of a specific implementation plan based on the initial diagnostic checklist (as undertaken in REACH) and could monitor fidelity to planned facilitation activities (e.g., by using the time-motion tracker adapted by BHIP, 31) may be of value for implementation and evaluation teams, but would need to provide sufficient flexibility to allow iterative adaptation to a changing context especially in longer term projects. Finally, further work needs to explore, in detail, how users of i-PARIHS are enacting facilitation and why they are enacting it in these particular ways, in order for us to develop targeted and useful resources to guide i-PARIHS-based implementation efforts.

## Conclusion

This exploratory co-design study examines how current users of i-PARIHS are operationalising the framework, which existing tools they are using, and where they have needed to develop new tools to best utilise the framework. This study highlights the value of existing tools from the Facilitation Guide [7] and provides a starting point to further refine and add to these tools within a Mi-PARIHS suite of resources. Our findings suggest that the *Facilitation Checklist* and *Facilitator’s Toolkit* can be adapted for a range of projects and can be used within pre-implementation planning, implementation and evaluation phases. It may be useful to add more explicit guidance and/or tools for using findings from the *Facilitation Checklist* within a structured implementation plan, and monitoring fidelity to the implementation plan, including recording how strategies are tailored to an evolving context.

Through this study, we have provided a detailed overview of how different implementation projects have used the i-PARIHS framework and have outlined their process of operationalising the framework which often goes unpublished. This study can help clinicians and novice researchers and first-time users to become more familiar with how an implementation project using i-PARIHS tools in practice and can serve as a useful road map for those who wish to use i-PARIHS within their own implementation efforts. Finally, this study suggests that i-PARIHS has begun to meaningfully address the criticisms of the preceding PARIHS framework relating to inadequate detail and support to operationalise the framework [4, 7] and identifies areas for further development of support and resources, particularly in the monitoring phase of implementation.

## Data Availability

Not applicable.

## Abbreviations

i-PARIHS: integrated-Promoting Action on Research Implementation in Health Services
Mi-PARIHS: Mobilising Implementation of i-PARIHS
CFIR: Consolidated Framework for Implementation Research
BHIP: Behavioural Health Interdisciplinary Program
CHERISH: Collaboration for Hospitalised Elders Reducing the Impact of Stays in Hospital
SIMPLE: A Systematised, Interdisciplinary Malnutrition Pathway for impLementation and Evaluation in hospitals
REACH: The Researching Effective Approaches to Cleaning Hospitals study
